# Major Depressive Disorder in French Caregivers Participating in the Profamille Psychoeducational Program

**DOI:** 10.1192/j.eurpsy.2025.2144

**Published:** 2025-08-26

**Authors:** Y. Hode, W. Hikmat, N. Guillard-Bouhet, J. Attal, R. Padovani, M.-C. Bralet, O. Canceil, M. Biotteau, I. Chereau-Boudet, C. Roussel, A. Montagne-Larmurier, S. Fattah, D. Willard

**Affiliations:** 1 Research department, Profamille organization, Chatenois, France; 2 Psychiatric Hospital of Kelaa Sraghna, Kelaa Sraghna, Morocco; 3 CREATIV Centre de REhabilitation et d’Activités Thérapeutiques Intersectoriel de la Vienne, Centre Hospitalier Henri Laborit, Poitiers; 4 Centre Hospitalier Universitaire de Montpellier, Montpelllier, France; 5 Independent Researcher, Le Tampon, Réunion; 6 CRISALID-HDF, Etablissement Public de Santé Mentale Oise, Clermont de l Oise, Clermont de l Oise; 7 Unit Research 1247 GRAP, NSERM, Amiens; 8 Fondation Santé des Etudiants de France, Paris; 9 Psychiatry, CHU de Tours, Tours; 10 Centre Expert Schizophrenie, CHU de Clermont Ferrand, Clermont-Ferrand; 11 Psychiatrie, Centre Hospitalier Annecy Genevois, Metz-Tessy; 12 Psychiatrie, CHU de Caen, Caen; 13 family intervention, World association of Social psychiatry, Mulhouse; 14 Pôle PEPIT, Groupe Hospitalier Universitaire Paris psychiatrie et neurosciences, Paris, Paris, France

## Abstract

**Introduction:**

Numerous studies indicate that caregivers of patients with schizophrenia experience significant burden and exhibit depressive symptoms as measured by the CES-D scale. Specific psychoeducational programs like the multifamilal CBT based Profamille program may help alleviate these symptoms. However, it is yet to be established the extent to which these caregivers meet the criteria for clinical depression as defined by the DSM-5, as well as whether the interventions employed in this program can effectively reduce the prevalence of depressive disorder among caregivers.

**Objectives:**

Assess the prevalence of major depressive disorder among caregivers at the start and end of the Profamille psychoeducational multifamily program.

**Methods:**

Caregivers were referred to the program by family organizations, welfare services or healthcare providers, or found out about the program through the local press.

A group consists of twelve caregivers of approximately ten patients, participating in 14 structured weekly sessions of Profamille program version V3.2, each lasting 4 hours. These sessions provide information about schizophrenia, develop coping strategies for caregivers, and employ behavioral and cognitive techniques to address depressive symptoms.

Mood was assessed using the PHQ-9, a self-report questionnaire consisting of nine items aligned with diagnostic criteria for major depressive disorder. Responses were analysed using an algorithm based on DSM-5 criteria to classify participants as having or not having major depressive disorder.

Given the paired nominal data, McNemar’s test was employed for analysis

**Results:**

A total of 507 caregivers were recruited, including 349 women. The average age of the participants was 58.0 years (SD = 9.0). At the beginning 14.6% of participants had a diagnosis of major depressive disorder. Fourteen sessions later this rate decreased to 5.7% (McNemar’s test, p-value < 0.00001)

**Conclusions:**

The annual prevalence of depression in the general population is approximately 5%, indicating a threefold over-representation among caregivers participating in the program. This rate of over-representation is consistent with other studies utilizing the CES-D, which also captures subclinical depression. Given the implications of depression for participants’ physical health, this underscores the need for systematic investigations aimed at providing support. The Profamille program, which employed specific cognitive behavioral techniques in a group setting, resulted in a significant reduction in depression rates over 14 sessions, bringing the final rates more in line with those of the general population. These findings suggest that the program effectively normalized the prevalence of depression. However, the absence of a control group limits our ability to assess the natural progression of depressive symptoms without program participation

**Disclosure of Interest:**

None Declared

